# Congenital Abnormalities Causing Hematocolpos: A Pictorial Essay

**DOI:** 10.5334/jbsr.3660

**Published:** 2024-09-24

**Authors:** Louise Dorthu, Denis Danthine

**Affiliations:** 1 Intern in Radiology, Radiology Department, CHU de Liège, Belgium; 2 Department of Radiology, University Hospital of Liège, Liège, Belgium

**Keywords:** hematocolpos, imperforate hymen, distal vaginal agenesis, transverse vaginal septum, OHVIRA

## Abstract

Hematocolpos, characterized by the accumulation of menstrual blood in the vagina, is a rare condition often misdiagnosed due to its uncommon occurrence and non-specific symptoms. Main causes include imperforate hymen, obstructed hemivagina with ipsilateral renal anomaly (OHVIRA), congenital lower vaginal atresia, and complete transverse vaginal septum.

Without early diagnosis and treatment, complications such as tubal adhesion, pelvic endometriosis, and infertility can occur.

This article reviews the differential diagnosis and treatment of hematocolpos.

## Introduction

### Embryology

Female genital tract originates from the Müllerian ducts. The cranial part forms the fimbria, the middle part forms the fallopian tube and the broad ligament, and the caudal parts fuse at the midline to form the utero-vaginal canal, which give rise to the uterus and the upper part of the vagina. The lower part of the vagina originates from the sinusal tubercle [1].

### Hematocolpos

Hematocolpos is a medical condition in which menstrual blood or secretory fluid accumulates in the vagina due to vaginal obstruction.

It stems from acquired vaginal occlusion or mostly congenital anomalies such as imperforate hymen, distal vaginal agenesis, complete transverse vaginal septum, and obstructed hemivagina with ipsilateral renal anomaly (OHVIRA), with imperforate hymen causing up to 90% of cases [2].

The main symptoms are periodic abdominal pain and primary amenorrhea.

Hematocolpos can also result in urinary tract obstruction, leading to urinary retention and acute renal infection.

Late complications include tubal infection, adhesion, pelvic endometriosis, infertility, and renal failure due to hydronephrosis [2].

Diagnosis involves clinical examination, ultrasonography, computed tomography scans, and magnetic resonance imaging (MRI), with MRI providing detailed soft tissue contrast [3].

### Müllerian duct anomaly classification

**Figure 1:**
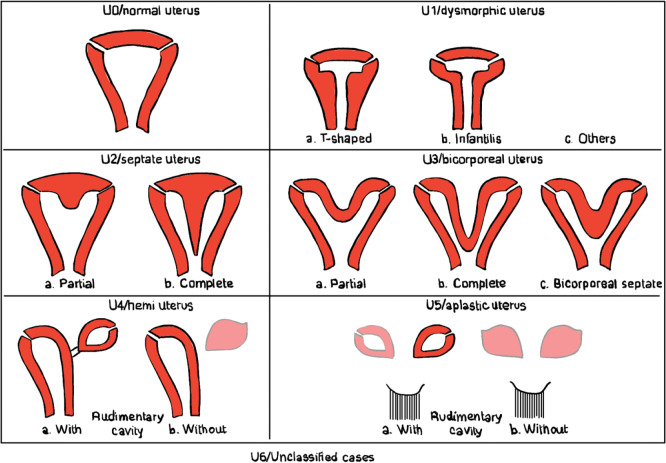
Schematic drawing of the ESHRE/ESGE classification. Inspired from Ref [5].

Buttram and Gibbons’ 1979 classification of Müllerian duct anomalies, modified by the American Society for Reproductive Medicine in 1988, was further refined by the European Society of Human Reproduction and Embryology (ESHRE) and the European Society for Gynecologic Endoscopy (ESGE) in 2013. This system categorizes uterine anomalies based on uterine wall thickness, coexisting cervical and vaginal anomalies (Figure 1) [4, 5].

## Cases

### Imperforate hymen

**Figure 2:**
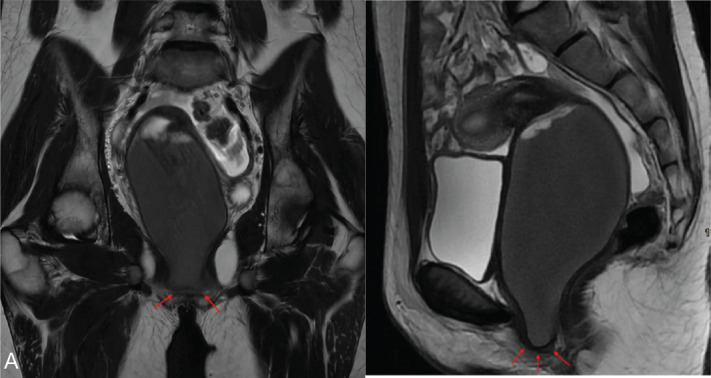

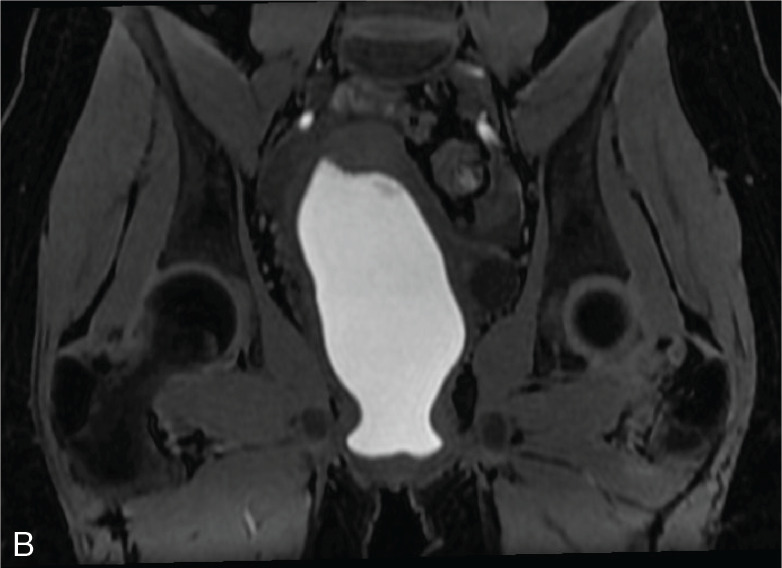

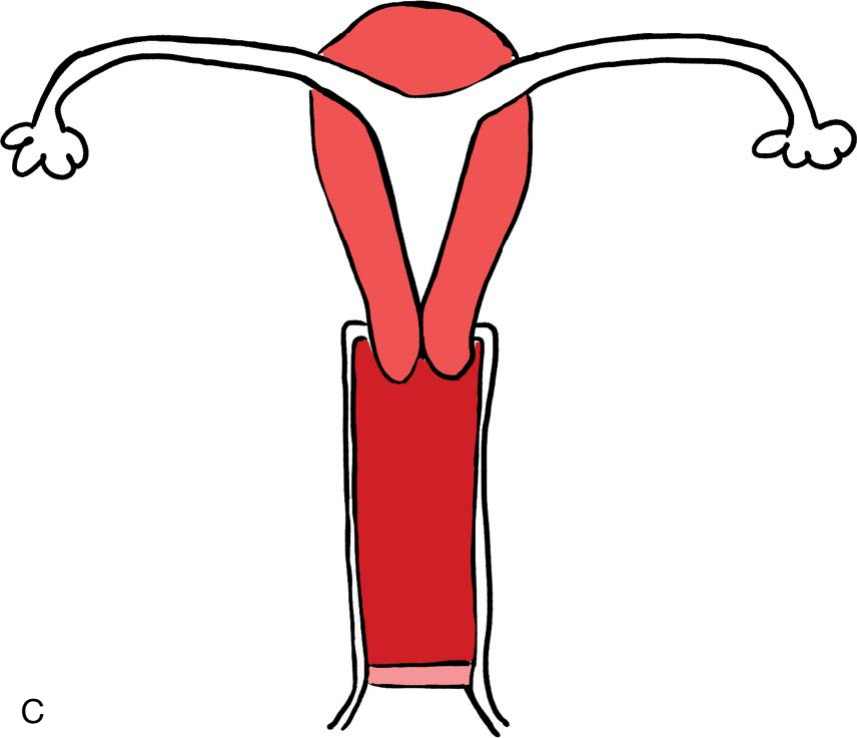
Imperforate hymen. **A.** Coronal and sagittal T2-weighted image (WI) with imperforate hymen (arrows) and fluid accumulation. **B.** Corresponding T1-weighted DIXON water image with hemorrhagic signal. **C.** Corresponding schematic representation. Inspired from Ref [2].

The most common cause of hematocolpos, imperforate hymen (incidence <1%), typically presents at puberty with symptoms of absent menstruation and hematocolpos. Treatment is surgical and involves hymenectomy or hymenotomy (Figure 2) [6].

### OHVIRA syndrome

**Figure 3:**
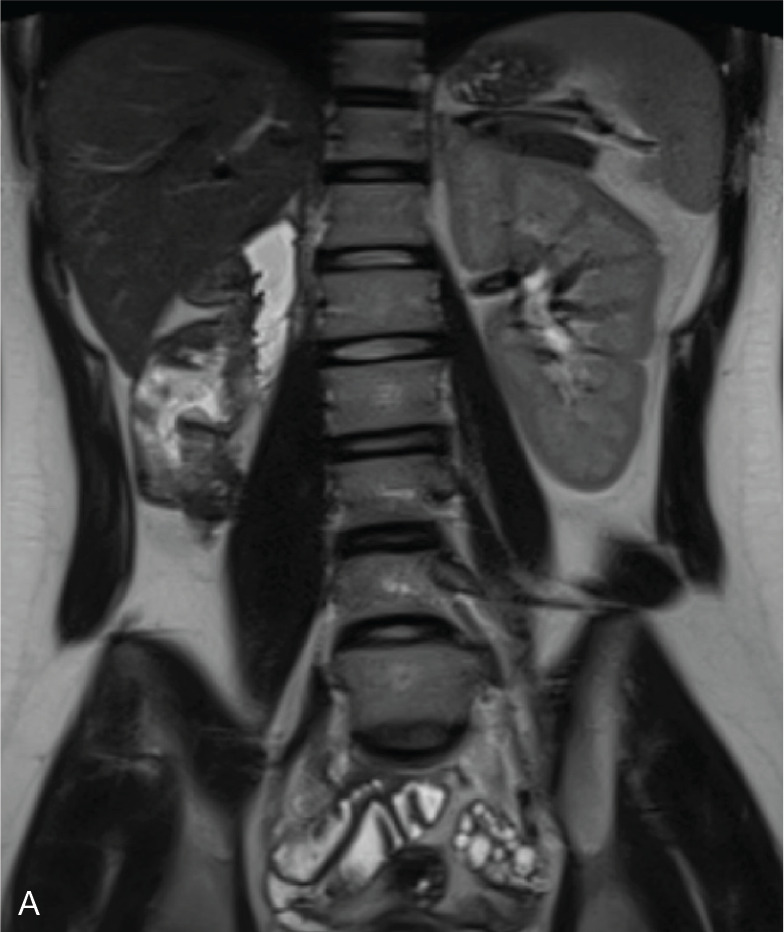

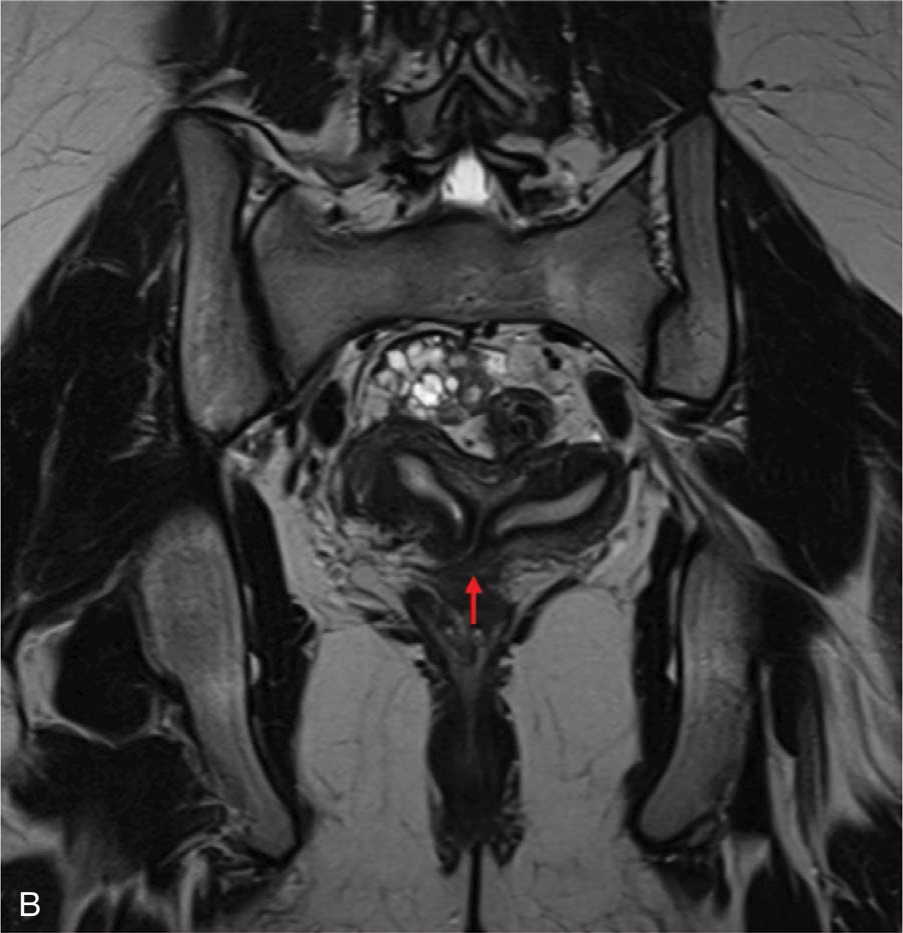

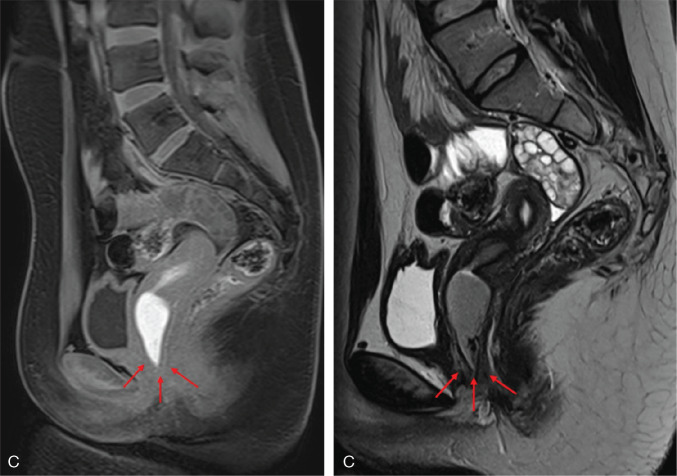

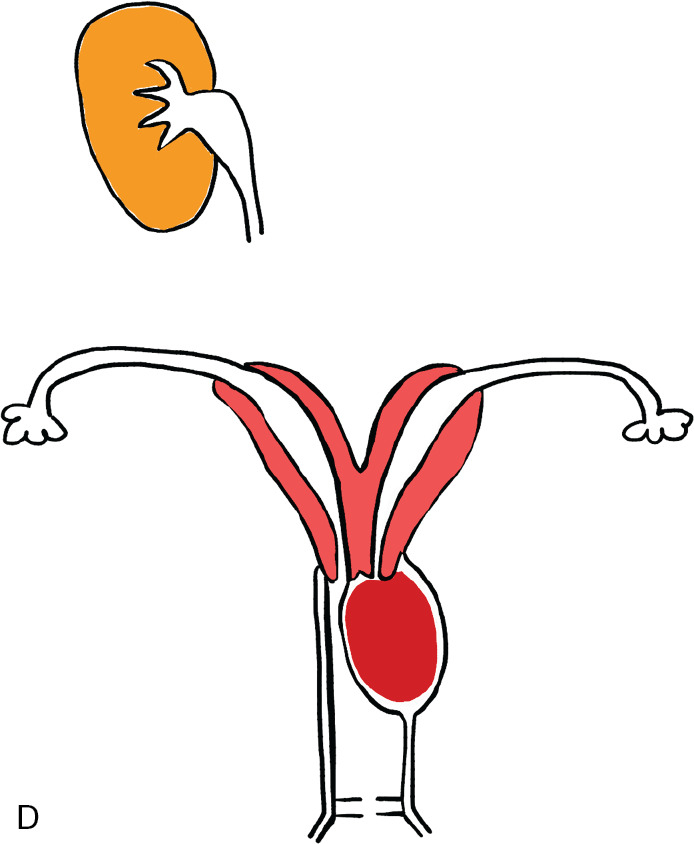
OHVIRA syndrome with uterus U3. **A.** Coronal T2 haste WI showing right renal agenesis. **B.** Coronal T2 WI showing uterus U3 (arrow). **C.** Sagittal T2 WI (right) and T1 WI dixon water (left) showing an obstructed hemivagina with hemorrhagic signal (arrows). **D.** Corresponding schematic representation. Inspired from Ref [2].

**Figure 4:**
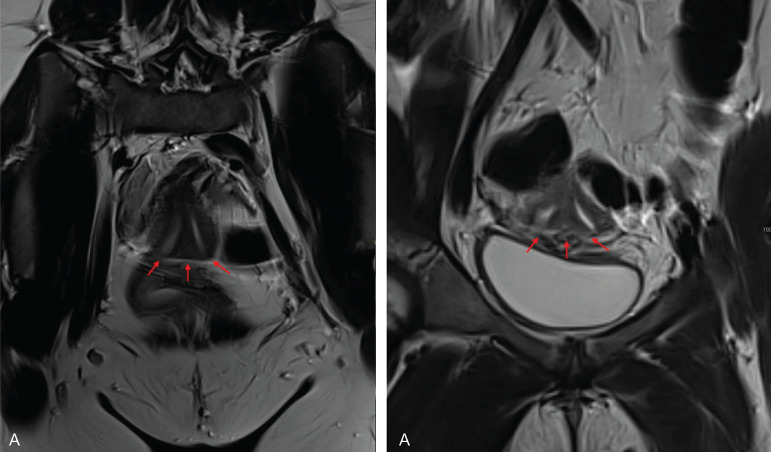

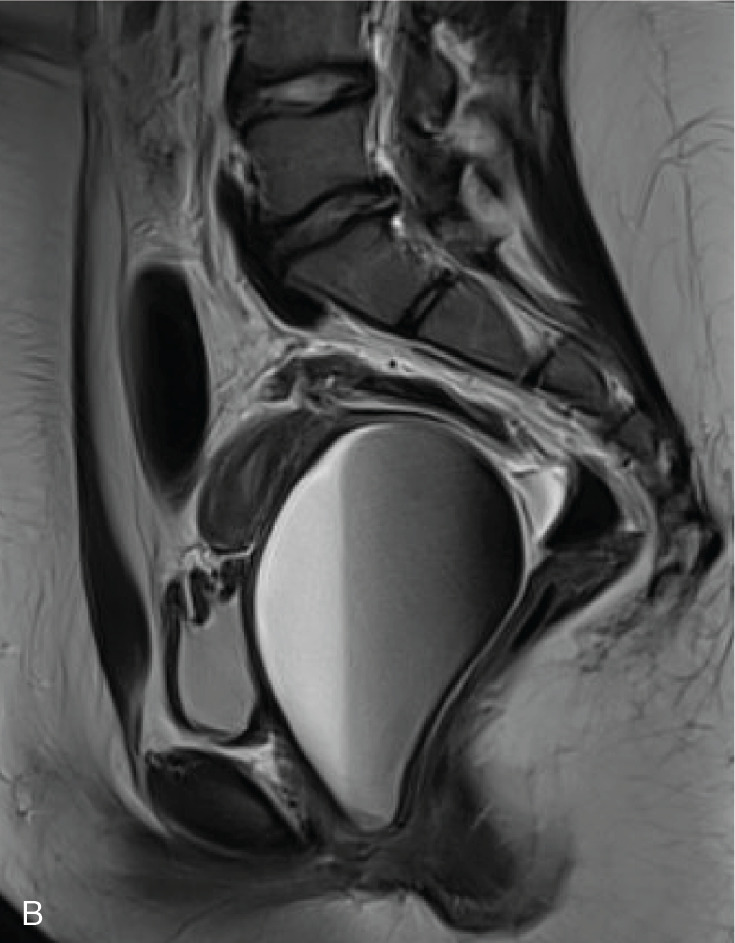

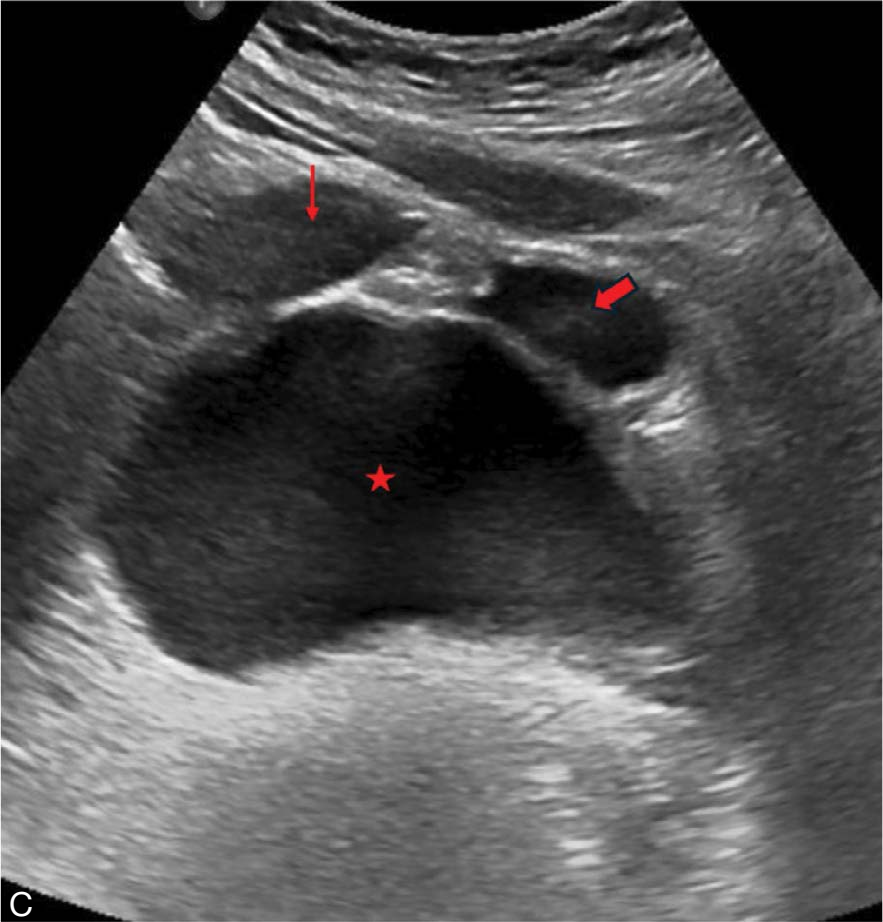

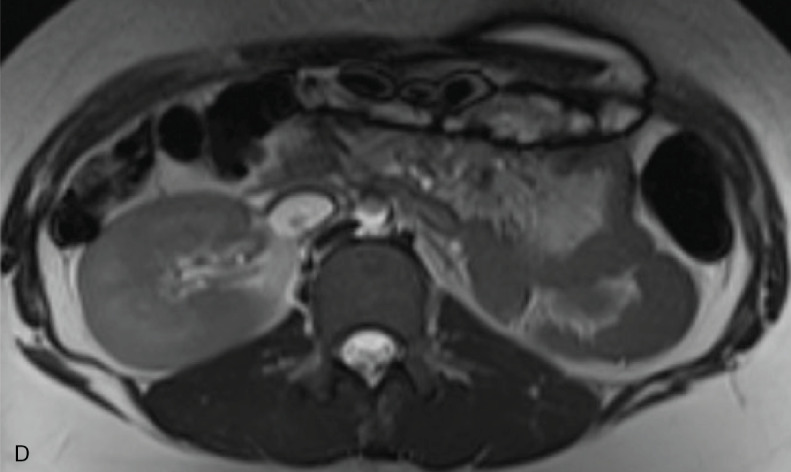

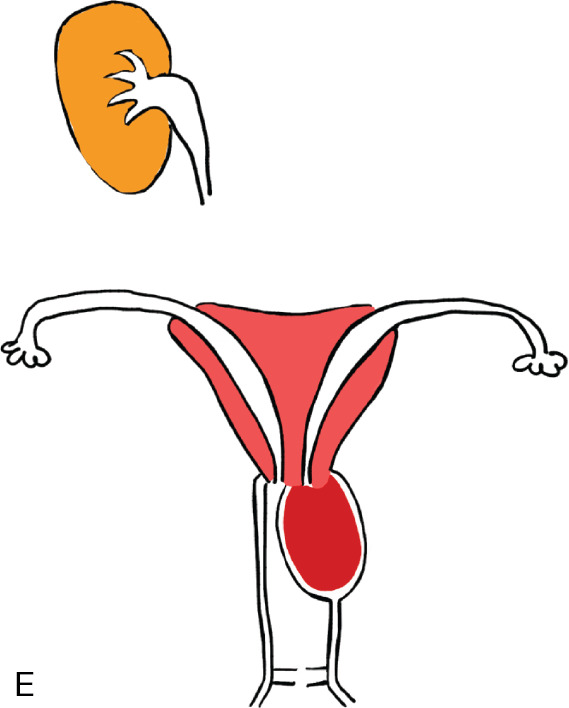
OHVIRA syndrome with uterus septate. **A.** Coronal (left) and oblique (right) T2 WI showing the uterus septate (arrows). **B.** Sagittal T2 WI with distended vagina. **C.** Corresponding US showing distended vagina (star), uterus (arrow), and bladder (short arrow). **D.** Axial T2 HASTE WI showing left kidney agenesis. **E.** Corresponding schematic representation. Inspired from Ref [2].

OHVIRA syndrome, or Herlyn–Werner–Wunderlich syndrome, is characterized by a didelphic uterus (bicornis bicervical uterus U3), obstructed hemivagina, and ipsilateral renal agenesis. It results from the abnormal development of the Müllerian and Wolffian ducts. Uterine anomalies, primarily the didelphic uterus (U3) (Figure 3), can also include bicornuate and septate uteri (Figure 4). Unilateral obstruction is commonly due to an oblique vaginal septum.

Ipsilateral renal agenesis is classical, although other renal anomalies are possible [7, 8].

### Distal vaginal atresia and transverse vaginal septum

Distal vaginal atresia and transverse vaginal septum involve segmental vaginal obstruction, with controversial terminology and embryology. Transverse vaginal septum can occur at any vaginal level but is most common in the upper and middle thirds.

While some view distal vaginal atresia as a form of thick transverse septum, others consider them distinct conditions.

**Figure 5:**
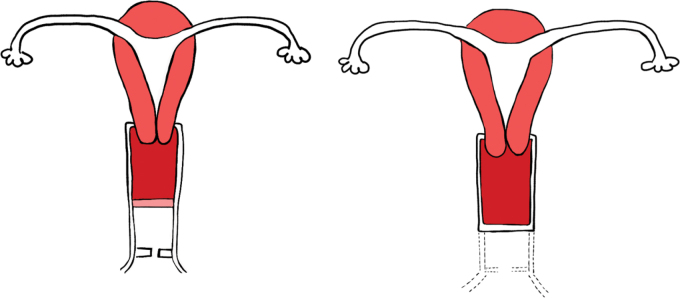
Vaginal septum and vaginal agenesia. Schematic drawing of vaginal septum (left) and vaginal agenesia (right). Inspired from Ref [2].

Both typically present similarly and are not associated with other urogenital anomalies (Figure 5) [9].

## Conclusion

Hematocolpos results from menstrual blood accumulation due to vaginal obstruction, mainly from congenital causes. Accurate differential diagnosis requires clinical examination, ultrasonography, and MRI. Early diagnosis and surgical treatment are crucial to prevent complications and preserve fertility.
